# A Natural Variant of Obestatin, Q90L, Inhibits Ghrelin's Action on Food Intake and GH Secretion and Targets NPY and GHRH Neurons in Mice

**DOI:** 10.1371/journal.pone.0051135

**Published:** 2012-12-10

**Authors:** Rim Hassouna, Philippe Zizzari, Odile Viltart, Seung-Kwon Yang, Robert Gardette, Catherine Videau, Emilio Badoer, Jacques Epelbaum, Virginie Tolle

**Affiliations:** 1 UMR-S 894 INSERM, Centre de Psychiatrie et Neurosciences, Université Paris Descartes, Sorbonne Paris Cité, Paris, France; 2 UMR 837 INSERM, Laboratoire “Développement et Plasticité du Cerveau Postnatal”, Centre de Recherches JPARC, Lille and Université Lille Nord de France (USTL- Lille 1), Lille, France; 3 School of Biomedical Sciences, The University of Queensland, Skerman Building (65), St Lucia, Queensland, Australia; 4 School of Medical Sciences and Health Innovations Research Institute, RMIT University, Bundoora, Melbourne, Victoria, Australia; CRCHUM-Montreal Diabetes Research Center, Canada

## Abstract

**Background:**

Ghrelin and obestatin are two gut-derived peptides originating from the same ghrelin/obestatin prepropeptide gene (*GHRL*). While ghrelin stimulates growth hormone (GH) secretion and food intake and inhibits γ-aminobutyric-acid synaptic transmission onto GHRH (Growth Hormone Releasing Hormone) neurons, obestatin blocks these effects. In Humans, *GHRL* gene polymorphisms have been associated with pathologies linked to an unbalanced energy homeostasis. We hypothesized that one polymorphism located in the obestatin sequence (Q to L substitution in position 90 of the ghrelin/obestatin prepropeptide, rs4684677) may impact on the function of obestatin. In the present study, we tested the activity of native and Q90L obestatin to modulate ghrelin-induced food intake, GH secretion, cFos activity in GHRH and Neuropeptide Y (NPY) neurons and γ-aminobutyric-acid activity onto GHRH neurons.

**Methodology/Principal findings:**

Food intake, GH secretion and electrophysiological recordings were assessed in C57BL/6 mice. cFos activity was measured in NPY-Renilla-GFP and GHRH-eGFP mice. Mice received saline, ghrelin or ghrelin combined to native or Q90L obestatin (30 nmol each) in the early light phase. Ghrelin stimulation of food intake and GH secretion varied considerably among individual mice with 59–77% eliciting a robust response. In these high-responders, ghrelin-induced food intake and GH secretion were reduced equally by native and Q90L obestatin. In contrast to *in vivo* observations, Q90L was slightly more efficient than native obestatin in inhibiting ghrelin-induced cFos activation within the hypothalamic arcuate nucleus and the nucleus tractus solitarius of the brainstem. After ghrelin injection, 26% of NPY neurons in the arcuate nucleus expressed cFos protein and this number was significantly reduced by co-administration of Q90L obestatin. Q90L was also more potent that native obestatin in reducing ghrelin-induced inhibition of γ-aminobutyric-acid synaptic transmission onto GHRH neurons.

**Conclusions/Significance:**

These data support the hypothesis that Q90L obestatin partially blocks ghrelin-induced food intake and GH secretion by acting through NPY and GHRH neurons.

## Introduction

Ghrelin is a 28 amino acid peptide principally synthesized in the stomach and was originally described as the endogenous ligand of the Growth Hormone Secretagogue 1a Receptor (GHS-R1a) [1], [2]. Ghrelin is the only orexigenic gastrointestinal peptide and one of its main functions is to stimulate growth hormone (GH) secretion [3], [4]. Binding of ghrelin to the GHS-R1a, which relays most of ghrelin's biological effects, is made possible due to a post-translational acylation on its serine in position 3 [5], [6]. GHS-R1a is highly expressed in the arcuate nucleus (ARC) of the hypothalamus, a key region involved in the control of GH secretion and appetite but also in the brainstem that receives information from gut vagal afferents [7], [8]. Within the hypothalamus, ARC Neuropeptide Y (NPY) and Growth Hormone Releasing Hormone (GHRH) neurons express the GHS-R1a [9], [10], and are a well-characterized target for ghrelin or GHS actions [11]–[13]. More recently, obestatin, a 23 amino acid peptide, derived from the cleavage of preproghrelin was discovered [14] and reported as an anorexigenic peptide ligand of the orphan receptor, GPR39, but these findings are controversial [15]–[18]. Nevertheless, when co-administered with ghrelin at equimolar doses, obestatin counteracts, for instance, ghrelin induced food intake and GH secretion in rodents [19]. The mechanism of action of obestatin and its interaction with ghrelin in the central nervous system remain poorly understood. The effect of obestatin on ghrelin-induced GH secretion is not mediated at the pituitary level [14], [19], suggesting that the interaction between ghrelin and obestatin is mainly mediated within the central nervous system. Indeed, recently, it was reported that obestatin blocks ghrelin-induced inhibition of γ-aminobutyric acid (GABA) synaptic transmission onto GHRH neurons [20].

In humans, ghrelin/obestatin prepropeptide gene (*GHRL*) polymorphisms have been associated with pathologies linked to an unbalanced energy homeostasis like anorexia or bulimia nervosa [21], [22]. One of those single nucleotide polymorphisms (SNP) (Q to L substitution in position 90 of preproghrelin, rs4684677) is located in the sequence encoding obestatin and anorexic patients carrying this SNP have a lower minimum body mass index (BMI) [23]. This polymorphism may thus influence the function of obestatin.

In this study, we compared the effect of native and Q90L obestatin in modulating ghrelin-induced food intake and GH secretion. These two parameters were measured after co-administration of the peptides in the light phase in *ad libitum* fed C57BL/6 mice. To define the central sites of interaction of these peptides, we assessed neuronal activation after co-administration of ghrelin and native or Q90L obestatin, in two key regions involved in regulation of GH secretion and/or food intake: the ARC in the hypothalamus and the NTS in the brainstem. Moreover, we investigated whether ghrelin and obestatin interacted directly on ARC NPY and GHRH neurons which relay ghrelin effects on food intake and GH release respectively [13], [24]–[29].

## Results

### Inter-individual variations in the effects of ghrelin, or ghrelin combined with native (hOb) and Q90L obestatin (hObQ90L) to modulate food intake and GH secretion

**Figure 1:**
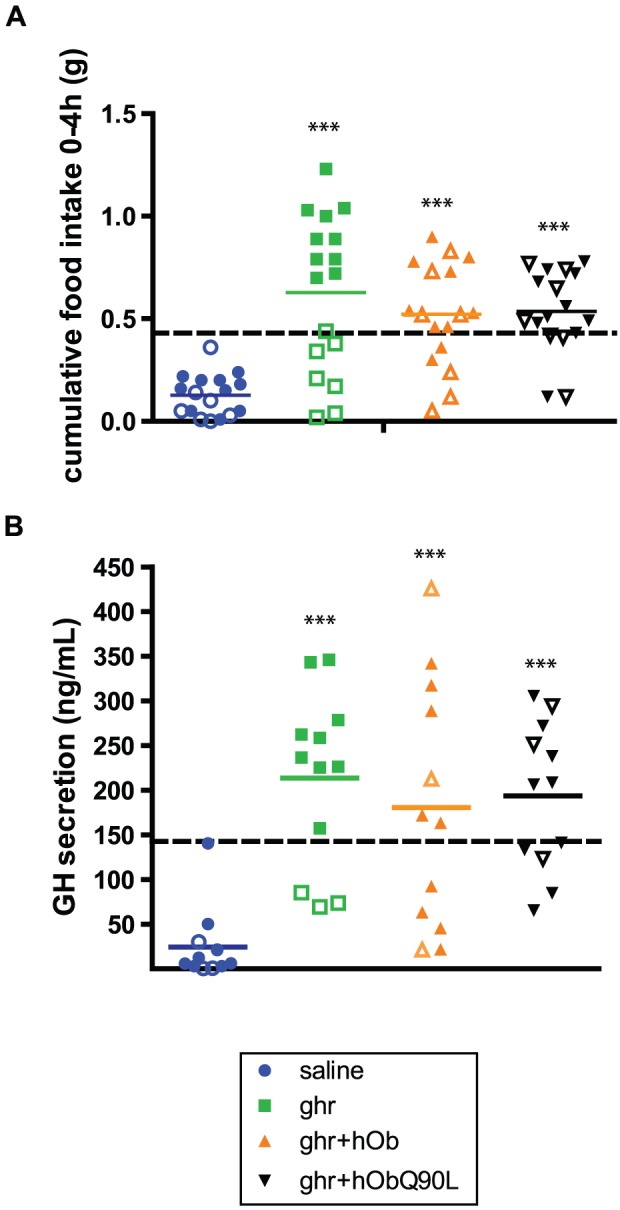
Differential effect of ghrelin on food intake and GH secretion in high and low-responders. (A) Cumulative 0–4 h food intake and (B) GH peak of secretion in individual mice injected i.p with saline (circles), 30 nmol of ghr (squares), ghr+hOb (triangles) or ghr+hObQ90L (inverted triangles) (30 nmol). 4 hours after ghrelin injection, 59% of mice increased their food consumption over a threshold of 0.42 g (high responders, closed symbols) whereas 41% of mice did not (low responders, open symbols) responders. The dotted line represents the threshold for ghrelin response. Bar represents mean of data. ***P<0.0001 *vs* saline.

The ability of human obestatin (hOb) and hObQ90L to inhibit ghrelin-induced food intake and GH secretion was tested after administration of equimolar doses (30 nmol ip) of ghrelin and hOb or hObQ90L during the light period in male C57BL/6 mice (Figure 1). We observed a high variability in the responses to peptides injections in individual mice and only a proportion responded to stimulation by ghrelin. Based on these observations, we determined a threshold to classify ghrelin-treated animals into either high or low responders. This was possible because each mouse was injected with each treatment in a cross-over designed manner. For food consumption, the threshold was defined as the mean value+3 standard deviations (SD) measured during 0–4 h after saline injection. For GH secretion, the threshold was defined as the mean value+3 standard deviations (SD) of the peak value recorded.

Fifty nine percent of mice increased their food consumption over a threshold of 0.42 g (high responders) 4 hours after ghrelin injection whereas 41% of mice did not (low responders) (Figure 1A). For GH secretion, 75% of mice exceeded the calculated threshold of 143 ng/ml (high responders) (Figure 1B). After co-administration of ghr+hOb or ghr+hObQ90L, the profile of the distribution of the responses was similar between high and low responders (Figure 1A and B). There was no difference between the two forms of obestatin in reducing the ghrelin-induced food intake or GH secretion when all animals were included.

Based on the above observations, the results for food intake and GH secretion were analyzed separately in high and low responders, the latter being shown in the Supporting Information.

### Native (hOb) and Q90L obestatin (hObQ90L) reduce ghrelin-induced food intake in high responders

**Figure 2:**
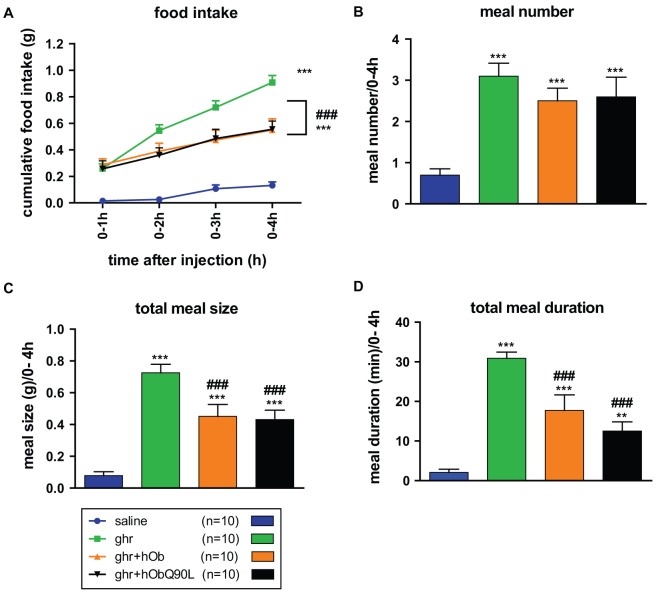
Effect of native (hOb) and Q90L obestatin (hObQ90L) on ghrelin-induced (Ghr) cumulative food intake and meal pattern in high responders C57BL/6 mice. (A) Mean cumulative 4 h food intake after the different treatments in high responders. (B–D) Meal number, size and duration within 4 h following the injections in high responders. Data represent mean±SEM. **P<0.01 *vs* saline, ***P<0.001 *vs* saline, ###P<0.001 *vs* ghr.

Feeding behavior was monitored after Ghr and/or after co-administration of Ghr+hOb/hObQ90L in male C57BL/6 mice. In high responders only, hOb and hObQ90L attenuated the ghrelin-induced cumulative food intake within 4 hours following treatments and the activity of the two forms of obestatin was equivalent (Figure 2A). Within the first hour following the injection, however, neither hOb nor hObQ90L inhibited the effects of ghrelin.

The analysis of the meal pattern revealed that the inhibitory effect of both forms of obestatin was due to a decrease in meal size and duration (Figure 2B–D). The effect of hObQ90L appeared to be slightly more pronounced than hOb more particularly in reducing ghrelin's effect on total meal duration.

In low responders, although ghrelin did not elicit an increase in cumulative food consumption (Fgure 1A), mice did respond to co-administration of ghr+hOb/hObQ90L notably by increasing meal number, size and duration (Figure 1B–D). hOb or hObQ90L injected alone did not affect food intake or meal pattern (Data not shown).

### Native (hOb) and Q90L obestatin (hObQ90L) reduce ghrelin-induced GH secretion in high responders

**Figure 3:**
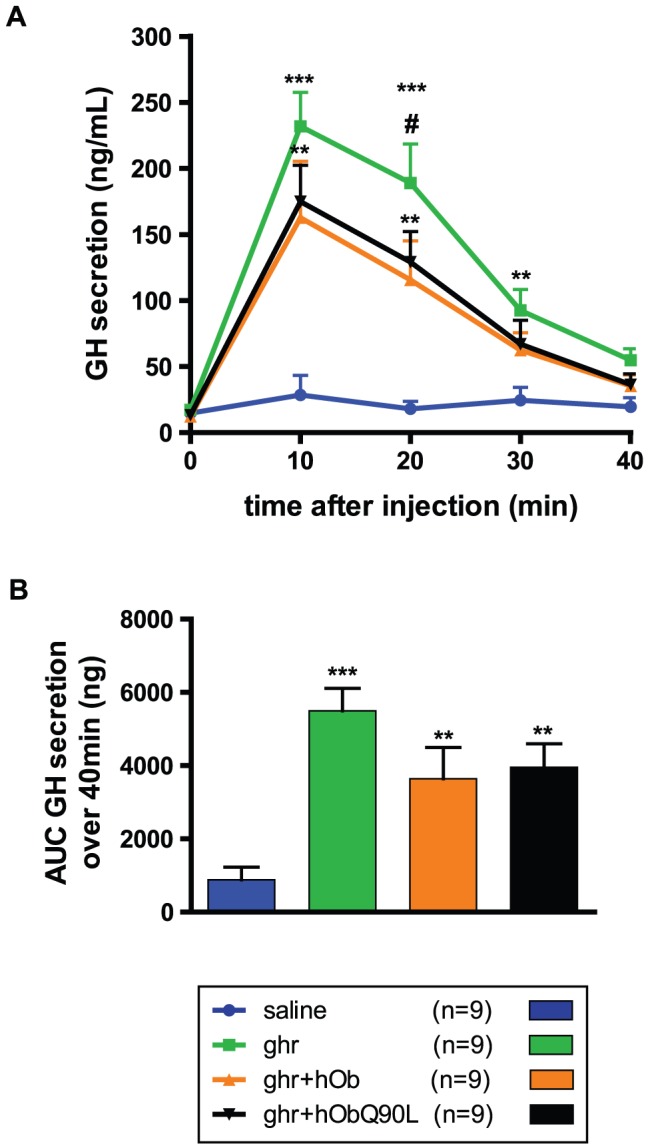
Effect of native (hOb) and Q90L obestatin (hObQ90L) on ghrelin-induced (Ghr) GH secretion in high responders C57BL/6 mice. (A) Mean GH secretion in response to the different treatments in high responders. (B) Area Under the curve of GH secretion over 40 minutes in high responders. Data represent mean±SEM. **P<0.01 *vs* saline, ***P<0.001 *vs* saline, #P<0.05 *vs* ghr+hOb.

The ability of hOb and hObQ90L to inhibit ghrelin-induced GH secretion was tested after administration of equimolar doses (30 nmol ip) of ghrelin and hOb/hObQ90L during the light period in both male and female mice (Figure 3). In high responders only, ghrelin induced an increase in the amplitude of GH secretion and this was significantly reduced by co-administration of Ghr+hOb but not Ghr+hObQ90L 20 minutes following the injection only (Figure 3A). Due to the inter-individual variability in all groups, the area under the curve (AUC) analysis showed a small inhibitory effect of both forms of obestatin although not statistically different from the ghrelin group (Figure 3B). There was no significant difference observed in the responses to the two forms of obestatin. In low responders, no statistical differences were observed between treatments (Figure 2).

### Q90L obestatin (hObQ90L) reduces ghrelin-induced cFos immunoreactivity in ARC and NTS

**Figure 4:**
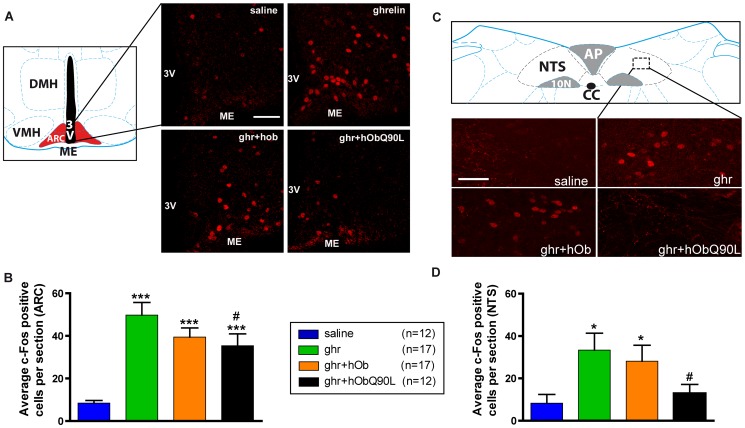
Effect of native (h/rOb) and Q90L obestatin (hObQ90L) on ghrelin-induced (Ghr) cFos immunoreactivity in C57BL/6 mice in the arcuate nucleus (ARC) and in the nucleus tractus solitarius (NTS). (A and C) Representative confocal photomicrophotographs of the cFos staining after each treatment in coronal sections at 1.9 mm of the interaural line in the ARC and −3.78 mm of the interaural line in the NTS ; (B and D) Average of cFos-positive nuclei per section after the different treatments between 2.2/1.6 mm of the interaural line for the ARC and between −3.78/−3.98 mm of the interaural line for the NTS. Data represent mean±SEM. ***P<0.001 *vs* saline, *P<0.05 *vs* saline, #P<0.05 *vs* ghr. Scale bar represents 100 µm in the ARC and 50 µm in the NTS. 3V: third ventricle, ME: median eminence, DMH: Dorsomedial Hypothalamic nucleus, VMH: Ventromedial Hypothalamic nucleus, ARC: Arcuate nucleus, NTS: Nucleus Tractus Solitarius, AP: Area Postrema, 10N: Dorsal Motor Nucleus of Vagus, cc: central canal.

cFos immunoreactive cells were quantified from 2.2/1.6 mm of the interaural line for the ARC and from −3.78/−3.98 mm of the interaural line for the NTS. Representative micrographs are illustrated on Figure 4A and C for ARC and NTS respectively. The increased number of cFos immunoreactive cells after ghrelin injection was significantly reduced by hObQ90L in both ARC (Figure 4B) and NTS (Figure 4D). The effects of both human (hOb) and rat (rOb) obestatin on cFos activation were pooled as no differences were observed between both forms (data not shown).

### Q90L obestatin (hObQ90L) reduces ghrelin-induced cFos immunoreactivity in ARC NPY neurons

**Figure 5:**
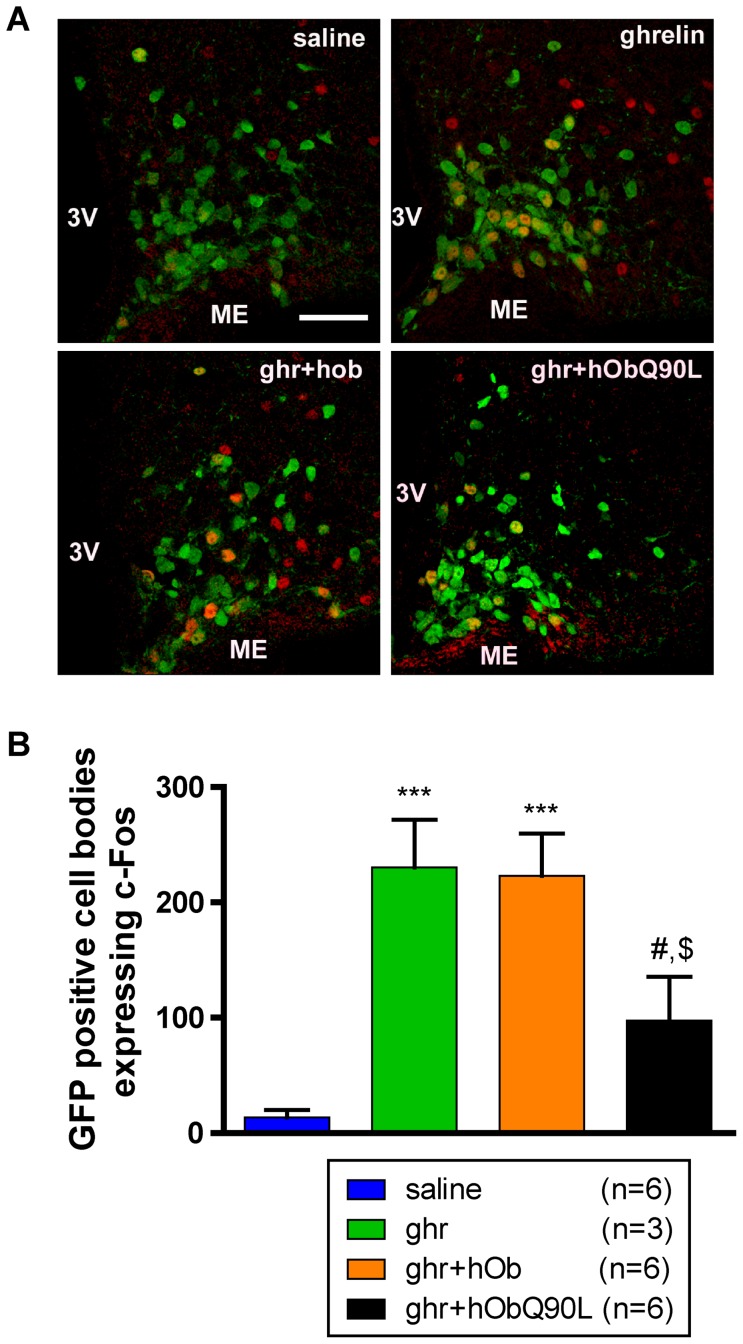
Effect of native (h/rOb) and Q90L obestatin (hObQ90L) on ghrelin-induced (Ghr) cFos immunoreactivity in arcuate nucleus (ARC) NPY neurons in C57BL/6 mice. (A) Representative confocal photomicrophotographs of hypothalamic coronal sections showing the merged images containing cFos staining in red and NPY-GFP cells in green after each treatment at 1.9 mm of the interaural line in the ARC. (B) Quantification of NPY-GFP positive cell bodies expressing cFos in the ARC after the different treatments. Data represent mean±SEM. ***P<0.001 *vs* saline, #P<0.05 *vs* ghr, $P<0.01 *vs* ghr+hOb. Scale bar represents 100 µm, 3V: third ventricle, ME: median eminence.

GFP positive cell number was quantified from 2.2/1.6 mm of the interaural line in the ARC. The number of GFP-positive neurons did not differ between the treatment groups (Figure 5A and Table S1). Ghrelin induced a significant increase in the number of NPY neurons expressing cFos compared to saline-injected animals (26% *vs* 2% of NPY neurons, respectively) (Figure 5B). In NPY neurons, co-localization with cFos was reduced significantly to 11% with hObQ90L but not with hOb (Figure 5A). Only 8% of GHRH neurons expressed cFos following ghrelin (Table S1). This was not significantly different from controls due to the lower proportion of ghrelin-activated neurons and the inter-individual variability.

### Native (hOb) and Q90L obestatin (hObQ90L) inhibit the ghrelin-induced decrease in evoked GABA synaptic responses in GHRH neurons

**Figure 6:**
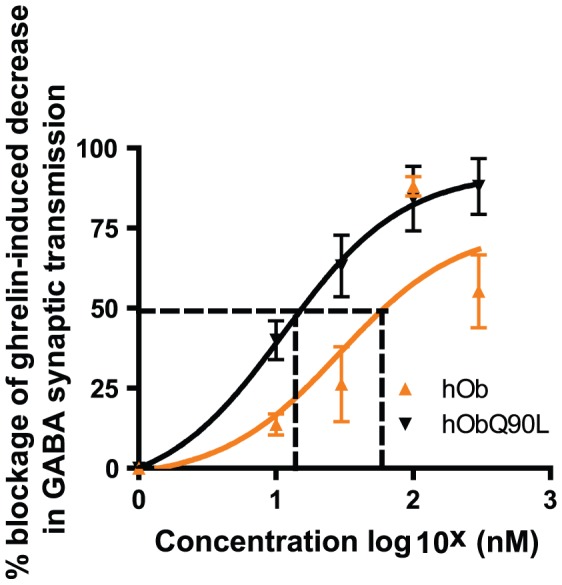
Effect of native (hOb) and Q90L obestatin (hObQ90L) on the ghrelin-induced (Ghr) decrease of evoked GABA synaptic responses in GHRH neurons. Dose response curves for hOb and hObQ90L (10, 30, 100 and 300 nM) represented on a semi-logarithmic scale. Ghrelin was applied at 300 nM. Data represent mean±SEM. The dotted line represents the EC50 values, 18 µM and 66.4 µM for hObQ90L and hOBhOb respectively. The two curves show significant differences (comparing best fit values of two groups using Student T-test, P<0.001).

Because ghrelin has been shown to reduce GABA synaptic transmission to GHRH neurons, evoked GABA synaptic responses were recorded in GHRH neurons expressing GFP. Both native and hObQ90L obestatin dose-dependently inhibited ghrelin effects on GABA transmission (Figure 6). hObQ90L obestatin was 2.5 times more potent in inhibiting the ghrelin-induced decrease of evoked GABA synaptic response in GHRH neurons (EC50 18 µM for hObQ90L and 66.4 µM for hOb).

## Discussion

In the present study, we observe that a natural variant of human obestatin, hObQ90L, is slightly more active than the native peptide to suppress some of ghrelin's biological effects in mice that respond to ghrelin. Indeed, only 59% and 75% of ghrelin-treated mice responded to ghrelin's actions on food intake and GH secretion, respectively. To our knowledge, this is the first report showing that i.p injection of ghrelin does not always lead to a major increase in food consumption or GH secretion. As the effects of obestatin can only be observed in high responders, inter-individual heterogeneity in ghrelin responses may explain why several studies failed to see any inhibitory action of obestatin on ghrelin-induced food intake or GH secretion [30], [31]. Controversy concerning the physiological significance or pharmacological actions of obestatin is partly due to the difficulty to reproduce published data and to the lack of concordant information in the literature [14], [15], [18], [30], [31]. Thus the pharmacological action of obestatin in reducing ghrelin-induced food intake and GH secretion [19] appears to depend upon the ability of individual animals to respond to ghrelin. For instance, ghrelin-induced cFos activation or feeding has been shown to be different in fed and fasted states [19], [32]. In the present study, we were unable to demonstrate any correlation between the food consumed within 15 minutes prior to peptides injections and ghrelin-induced feeding (R^2^ = 0,001, P = 0,8870). Thus, biological parameters, like nutritional status or stress level, could impact on the response to ghrelin but this remains to be demonstrated.

ObQ90L polymorphism is found in the general population with a frequency of 2–14%. Although this single nucleotide polymorphism does not appear to be associated with metabolic or eating disorders according to population studies [33]–[36], anorexic patients carrying this SNP have a lower minimum body mass index (BMI) [23]. Together with the current data, it may suggest that ObQ90L polymorphism, located in the sequence encoding for obestatin, has a stronger inhibitory action on some of the circuits regulating feeding compared to the natural peptide, which has been found to be a functional ghrelin antagonist previously [19], [20].

Although no significant difference between native and Q90L obestatin is observed on cumulative food intake over 4 hours, analysis of meal structure reveals that hObQ90L, at the dose of 30 nmol, has a slightly larger effect in reducing total meal duration than the natural peptide. Real differences in potencies between both peptides cannot be ascertained from analysis of feeding pattern as dose-responses were not performed. At any rate, the results indicate that native and Q90L obestatin partially reduce ghrelin-induced food intake by modulating satiation (i.e meal termination). A suppressive effect on the ghrelin-induced food intake is not observed within the first hour following the injection, suggesting that the inhibitory action of both native and Q90L obestatin on ghrelin-induced food intake is delayed. However in a previous study [19], the inhibitory effect of obestatin on the ghrelin-induced food intake was observed immediately following the injection. Such a discrepancy could be related to different experimental paradigms, injections being performed just before the dark phase in the previous study and during the light phase herein. For ghrelin-induced GH secretion, inhibition by hObQ90L is not more pronounced than native obestatin. In addition, we were unable to demonstrate an effect of obestatin *per se*, confirming most of previous data [22].

In the absence of an identified obestatin receptor and adequate tools to study obestatin function (agonists or antagonists), the mechanism of action of obestatin and its interaction with ghrelin in the central nervous system have remained poorly understood. We thus tested for the first time whether obestatin could modulate ghrelin effects by acting on the same neuronal populations synthesizing the orexigenic peptide NPY. ARC NPY neurons are a well-characterized target for ghrelin actions in the hypothalamus. In the rat, in-situ hybridization studies show co-localization between the GHS-R and NPY (and GHRH) mRNAs [9], [10]. In addition, intravenous administration of GHRP-6, a synthetic GHS, activates cFos in GHRH and NPY neurons: around half of the activated cells are NPY containing and around one quarter are GHRH containing cells [13]. Here, we describe for the first time by using NPY-Renilla-GFP mice that i.p administration of ghrelin activates 26% of NPY neurons in the ARC while 53% of cFos immunoreactive cells are GFP positive. This is consistent with previous results showing that a majority of cFos immunoreactive cells after i.p injection of ghrelin express NPY mRNA in mice [11]. The proportion of NPY neurons expressing cFos is robustly reduced from 26 to 11% by Q90L obestatin, demonstrating that the actions of this natural variant are relayed through this population of orexigenic neurons. In addition, the activation of NTS neurons, another target of ghrelin, is also more reduced by Q90L obestatin than with the native peptide. As the NTS relays information about satiety and satiation, these data are consistent with an effect of obestatin on ghrelin-induced increase in meal size and duration.

Only a small proportion of GHRH-eGFP neurons were activated by ghrelin treatment in the current study (8% of GHRH-eGFP neurons are cFos positive). Consequently, a significant inhibitory action of obestatin on GHRH neurons using cFos quantification could not be demonstrated, despite the fact that GHRH neurons express the GHS-R [9], [10] and GHRH does play a role in ghrelin-induced GH secretion in rats [24]. In addition, this contrasts with data obtained in rats showing that the synthetic GHSs, GHRP-6 and KP-102, activate cFos in a significant number of GHRH neurons, although the proportion of GHRH cells expressing cFos mRNA was very different with both GHS (38% with GHRP-6 and 20% with KP-102) [13], [37]. Thus differences in activated cells from one study to another may be due to species differences, measurement of mRNA versus GFP-positive cells or to differences in the mechanism of actions of various GHS-R ligands. A recent study from our laboratory using patch-clamp recording in mice demonstrated that ghrelin activates 44% of recorded GHRH neurons by inhibiting GABA synaptic transmission onto GHRH neurons and this was inhibited by obestatin [20]. The discrepancies between the cFos data and single-cell recording following ghrelin stimulation may be explained by differences in the experimental conditions and techniques used (electrically induced synaptic responses after direct application of ghrelin on hypothalamic slices versus cFos activation after peripheral ghrelin administration). Thus we compared the effects of native and Q90L obestatin using patch-clamp recording and demonstrated that Q90L obestatin blocks ghrelin-induced decreases in GABA synaptic transmission more potently than native obestatin. In contrast to the behavioral and physiological observations in mice, cellular assays appear to reveal a difference between native and Q90L obestatins.

In conclusion, hObQ90L not only retains its ability to reduce ghrelin effects on food intake and GH secretion, in mice that respond to ghrelin, but it also has a stronger effect than native obestatin in inhibiting neuronal activity in key target circuits regulating feeding or GH secretion. Substitution of one amino-acid by another has previously been shown to modify the biological activity of peptides [38], [39]. These results extend our knowledge about the mechanism of action of this functional ghrelin antagonist and are in keeping with clinical data showing that, in anorexic patients, this variant was more frequent amongst women with the lowest body mass index [23].

## Materials and Methods

### Ethics Statement

All experiments were carried out in accordance with the European Communities Council Directive (86/609/EEC) and were approved by the Animal Experimentation Committee of Paris Descartes University.

### Animals

Adult C57Bl/6 male mice (Charles River) (11 weeks old upon arrival) were housed in individual cages for the food intake experiments. Heterozygous GHRH-eGFP transgenic mice [40] and NPY-Renilla GFP transgenic male and female mice [41] were obtained by crossing transgenic to C57Bl/6 mice. The transgenic offspring were genotyped by PCR amplification of tail DNA. All animals were housed in a room under controlled illumination (7:00 AM to 7:00 PM) and temperature (22–24°C) and had free access to food and water. All experiments were started between 9:30 AM and 10:30 AM. Male and female mice were used for GH response and immunohistochemistry experiments and only males were used for the feeding and electrophysiological experiments. No sex differences were observed.

### Acute injections of peptides

For the food intake and GH secretion experiments, mice received an intraperitoneal (i.p) injection of saline, rat acyl-ghrelin (ghr), ghrelin+human obestatin (ghr+hob) and ghrelin+human Q90L obestatin (ghr+hobQ90L), 30 nmol each (NeoMPS Strasbourg, France). Experiments were repeated in a crossover designed manner so that each mouse received all treatments randomly separated by two days of washout. For the immunohistochemical experiments, separate groups of mice received an i.p injection of either saline, rat acyl-ghrelin, ghr+rat or human obestatin (r/hOb), or ghr+hobQ90L (30 nmol each). 90 minutes later the animals were anesthetized and perfusion-fixed (see below).

### Food intake monitoring and meal patterns analysis

Mice were habituated to single housing and to the automated drinking/feeding station (TSE systems, GmbH, Germany) for 1 week before the beginning of the experiment. On each experimental day, mice received an i.p injection of the peptides. Feeding behavior was recorded continuously for 24 hours after the injection by means of high precision sensors, attached to the top of the cage.

Meal patterns were analyzed using the following definition: a meal consists of the consumption of 0.03 g of food separated from the next feeding episode by at least 10 minutes (intermeal interval, [42]–[44]). For each mouse, the meal number, the total meal size (g) and the total meal duration (minutes) were measured for 4 hours following the injection of the peptides.

### Repeated blood samplings for GH assay

Mice were habituated to single housing before the beginning of the experiment and handled in order to minimize stress. 4 µl of the whole venous blood was obtained from the tail vein every ten minutes [45], for one hour after the i.p injection of the peptides. Blood samples were collected in 116 µl of the Enzyme ImmunoAssay (EIA) GH buffer (PBS-0.05% tween) and kept on ice until the end of experiment and then transferred to −20°C for storage before further analysis.

Whole blood GH concentrations were evaluated by EIA as previously described [45].

### Immunohistochemistry for cFos and co-localization studies

GHRH and NPY neurons were visualized using GFP fluorescence. Specific co-localization experiments between GHRH or NPY and cFos were performed as follows. Ninety minutes after i.p injection of the peptides, the mice were anaesthetized (5.47 mg/30 g BW of sodium pentobarbital) and perfused transcardially with 0.9% saline followed by fixative containing 4% paraformaldehyde (PFA) in PB (0,1 M). The brains were removed, post-fixed in 4% PFA in PB for 2 hours at room temperature and cryoprotected in 30% sucrose for 2 days at 4°C before freezing in 2-methyl-butane (at −35°C). Serial coronal sections, 25 µm thick, were cut using a freezing microtome (Frigomobile, Leica, Wetzlar, Germany). Immunofluorescence was performed on free-floating sections, separated by 100 µm for the entire rostro-caudal extent of the hypothalamic arcuate nucleus (ARC) and the nucleus solitary tract (NTS) of the brainstem. The sections were first incubated with primary antibody: anti-cFos raised in rabbit (1∶20000, Ab-5, Jackson Laboratories, West Grove, PA, USA) and then with Cy3 conjugated donkey anti-rabbit antiserum (1∶800, Jackson Laboratories, West Grove, PA, USA). Sections were examined using a Zeiss Axioplan microscope (Carl Zeiss, Le Pecq, France). The number of cFos positive cell nuclei and GFP positive cell bodies were counted bilaterally under a 40× magnification, in ARC (interaural line: 2.2 to 1.6 mm from Paxinos and Watson's mouse brain atlas, 1997) and in the NTS (interaural line: −3.78 to −3.98 mm). GFP positive cell bodies expressing cFos were visualized using a confocal SP5 microscope (Leica, Wetzlar, Germany). The number of cells showing co-localization was determined using the Image-J software (http://rsbweb.nih.gov/ij/) on series of continuous optical sections with 0.5 µm increment along the z-axis of the section under a 40× magnification. Confocal images presented were obtained from individual optical slices.

### Electrophysiological Experiments

Electrophysiological experiments were conducted as described previously [13]. Briefly, recordings were performed at room temperature on 200 µm thick hypothalamic coronal slices from 8–13 week-old male GHRH-eGFP transgenic CB57Bl/6J placed in a bath solution containing 124 mM NaCl, 3 mM KCl, 1.25 mM NaH_2_PO_4_, 1 mM MgSO_4_, 2 mM CaCl_2_, 26 mM NaHCO_3_, 10 mM Glucose, 10 µM CNQX (6-cyano-7nitroquinoxaline-2,3-dione, AMPA-R antagonist) and 50 µM D-AP-5 (2-amino-5-phosphonovalerate, NMDA-R antagonist), pH 7.3, 300–310 mOsm/L, and perfused at a rate of 2 ml/minutes. GHRH-eGFP ARC neurons were visually identified by fluorescent microscopy. To record electrically induced synaptic responses, patch recording pipettes were filled with 131 mM CH_3_O_3_SCs, 6 mM CsCl (intracellular Cs was used to block potassium channels), 2 mM Mg Cl_2_, 10 mM HEPES, 1.1 EGTA, 5 mM Lidocaine N-ethyl bromide (QX-314, to block sodium channels), 4 mM ATP-Mg, pH 7.3 (adjusted with 1 M CsOH) and 290–300 mOsm/L. Neurons were voltage-clamped at −20 mV. GABA synaptic responses were recorded using an Axopatch 1D amplifier (Axon Instruments, Union City, CA, USA), digitized using a Digidata 1200 interface (Axon Instruments) and stored on a computer. On-line analysis and storage of current data into data files performed using the pClamp 6.0.4 software from Axon Instruments. In all experiments, stimuli (0.2 msec duration, 0.3–1 mA intensity, 0.25 Hz) were applied to determine the postsynaptic response amplitudes and to measure the time-dependent effects of drugs. Drugs were diluted in the external medium to the desired concentration and applied by bath perfusion (ghrelin: 300 nM, hOb/hObQ90L: 10, 30, 100 and 300 nM). After acquisition of a 10 minutes baseline of current amplitudes, hOb and hObQ90L were applied for 5–6 minutes, followed by 10 minutes washing periods. Amplitudes of induced synaptic currents were normalized relative to the baseline prior to the application of drugs.

### Statistical analysis

Values are given as mean±SEM, and statistical analysis were performed using ANOVA, repeated measure ANOVA followed by a Fisher *post-hoc* test when the p value of the ANOVA was significant (P<0.05) using statview software (SAS institute Inc., Cary, NC, USA).

Areas Under the Curve (AUC) were calculated by the trapezoidal method.

The effects of the drugs in electrophysiological experiments were determined by comparing the best-fit values of two dose-response curves using Student T-test in the GraphPad PRISM 5 software (GraphPad Software, San Diego, CA, USA). The effect were different if P<0.05.

## Supporting Information

Figure S1 (A) Mean cumulative 4 h food intake after the different treatments in low responders. (B–D) Meal number, size and duration within 4 h following treatment in low responders. Data represent mean±SEM. *P<0.05 *vs* saline, **P<0.01 *vs* saline, #P<0.05 *vs* ghr.
(EPS)

Figure S2 (A) Mean GH secretion in response to the different treatments in low responders. (B) Area Under the curve of GH secretion over 4 h in low responders. Data represent mean±SEM.
(EPS)

Table S1 **Effect of native (h/rOb) and Q90L obestatin (hObQ90L) on ghrelin-induced (Ghr) cFos immunoreactivity in ARC NPY and GHRH neurons in C57BL/6 mice.**
(EPS)

## References

[1] Kojima M, Hosoda H, Date Y, Nakazato M, Matsuo H, et al (1999) Ghrelin is a growth-hormone-releasing acylated peptide from stomach. Nature 656–660. 10604470 10.1038/45230

[2] Tomasetto C, Karam SM, Ribieras S, Masson R, Lefèbvre O, et al (2000) Identification and Characterization of a Novel Gastric Peptide Hormone: The Motilin-Related Peptide. Gastroenterology 395–405. 10.1053/gast.2000.9371 10930375

[3] Tolle V, Zizzari P, Tomasetto C, Rio MC, Epelbaum J, et al (2001) In vivo and in vitro effects of ghrelin/motilin-related peptide on growth hormone secretion in the rat. Neuroendocrinology 54–61. 11174017 10.1159/000054620

[4] Tschop M, Smiley DL, Heiman ML (2000) Ghrelin induces adiposity in rodents. Nature 908–913. 11057670 10.1038/35038090

[5] Gutierrez JA, Solenberg PJ, Perkins DR, Willency JA, Knierman MD, et al (2008) Ghrelin octanoylation mediated by an orphan lipid transferase. Proceedings of the National Academy of Sciences 6320–6325. 10.1073/pnas.0800708105 PMC2359796 18443287

[6] Yang J, Brown MS, Liang G, Grishin NV, Goldstein JL (2008) Identification of the Acyltransferase that Octanoylates Ghrelin, an Appetite-Stimulating Peptide Hormone. Cell 387–396. 10.1016/j.cell.2008.01.017 18267071

[7] Guan XM, Yu H, Palyha OC, McKee KK, Feighner SD, et al (1997) Distribution of mRNA encoding the growth hormone secretagogue receptor in brain and peripheral tissues. Brain Res Mol Brain Res 48: 23–29. 9379845 10.1016/s0169-328x(97)00071-5

[8] Katayama M, Nogami H, Nishiyama J, Kawase T, Kawamura K (2000) Developmentally and regionally regulated expression of growth hormone secretagogue receptor mRNA in rat brain and pituitary gland. Neuroendocrinology 72: 333–340. 11146416 10.1159/000054602

[9] Willesen MG, Kristensen P, Rømer J (1999) Co-localization of growth hormone secretagogue receptor and NPY mRNA in the arcuate nucleus of the rat. Neuroendocrinology 306–316. 10567856 10.1159/000054491

[10] Tannenbaum GS, Lapointe M, Beaudet A, Howard AD (1998) Expression of growth hormone secretagogue-receptors by growth hormone-releasing hormone neurons in the mediobasal hypothalamus. Endocrinology 4420–4423. 9751527 10.1210/endo.139.10.6330

[11] Wang L, Saint-Pierre DH, Taché Y (2002) Peripheral ghrelin selectively increases Fos expression in neuropeptide Y - synthesizing neurons in mouse hypothalamic arcuate nucleus. Neuroscience Letters 47–51. 10.1016/s0304-3940(02)00241-0 12023064

[12] Dickson SL, Leng G, Robinson IC (1993) Systemic administration of growth hormone-releasing peptide activates hypothalamic arcuate neurons. Neuroscience 53: 303–306. 8492908 10.1016/0306-4522(93)90197-n

[13] Dickson SL, Luckman SM (1997) Induction of c-fos messenger ribonucleic acid in neuropeptide Y and growth hormone (GH)-releasing factor neurons in the rat arcuate nucleus following systemic injection of the GH secretagogue, GH-releasing peptide-6. Endocrinology 138: 771–777. 9003014 10.1210/endo.138.2.4907

[14] Zhang JV (2005) Obestatin, a Peptide Encoded by the Ghrelin Gene, Opposes Ghrelin's Effects on Food Intake. Science 996–999. 10.1126/science.1117255 16284174

[15] Chartrel N, Alvear-Perez R, Leprince J, Iturrioz X, Goazigo ARL, et al (2007) Comment on “Obestatin, a Peptide Encoded by the Ghrelin Gene, Opposes Ghrelin's Effects on Food Intake”. Science 766c–766c. 10.1126/science.1135047 17289961

[16] Holst B, Egerod KL, Schild E, Vickers SP, Cheetham S, et al (2006) GPR39 Signaling Is Stimulated by Zinc Ions But Not by Obestatin. Endocrinology 13–20. 10.1210/en.2006-0933 16959833

[17] Lauwers E, Landuyt B, Arckens L, Schoofs L, Luyten W (2006) Obestatin does not activate orphan G protein-coupled receptor GPR39. Biochemical and Biophysical Research Communications 21–25. 10.1016/j.bbrc.2006.09.141 17054911

[18] Annemie VD, Debby VD, Valentijn V, Bart DS, Walter L, et al (2009) Central administration of obestatin fails to show inhibitory effects on food and water intake in mice. Regulatory Peptides: Elsevier B.V. 77–82. 10.1016/j.regpep.2009.04.014 19422857

[19] Zizzari P, Longchamps R, Epelbaum J, Bluet-Pajot MT (2007) Obestatin partially affects ghrelin stimulation of food intake and growth hormone secretion in rodents. Endocrinology 1648–1653. 17204551 10.1210/en.2006-1231 PMC1890395

[20] Feng DD, Yang SK, Loudes C, Simon A, Al-Sarraf T, et al (2011) Ghrelin and obestatin modulate growth hormone-releasing hormone release and synaptic inputs onto growth hormone-releasing hormone neurons. European Journal of Neuroscience 732–744. 21777303 10.1111/j.1460-9568.2011.07787.x

[21] Ando T, Komaki G, Naruo T, Okabe K, Takii M, et al (2006) Possible role of preproghrelin gene polymorphisms in susceptibility to bulimia nervosa. Am J Med Genet 929–934. 16921495 10.1002/ajmg.b.30387

[22] Hassouna R, Zizzari P, Tolle V (2010) The ghrelin/obestatin balance in the physiological and pathological control of GH secretion, body composition and food intake. Journal of Neuroendocrinology 794–804. 10.1111/j.1365-2826.2010.02019.x 20456603

[23] Dardennes RM, Zizzari P, Tolle V, Foulon C, Kipman A, et al (2007) Family trios analysis of common polymorphisms in the obestatin/ghrelin, BDNF and AGRP genes in patients with Anorexia nervosa: Association with subtype, body-mass index, severity and age of onset. Psychoneuroendocrinology 106–113. 17197106 10.1016/j.psyneuen.2006.11.003

[24] Tannenbaum GS (2003) Interrelationship between the Novel Peptide Ghrelin and Somatostatin/Growth Hormone-Releasing Hormone in Regulation of Pulsatile Growth Hormone Secretion. Endocrinology 967–974. 10.1210/en.2002-220852 12586774

[25] Shintani M, Ogawa Y, Ebihara K, Aizawa-Abe M, Miyanaga F, et al (2001) Ghrelin, an endogenous growth hormone secretagogue, is a novel orexigenic peptide that antagonizes leptin action through the activation of hypothalamic neuropeptide Y/Y1 receptor pathway. Diabetes 227–232. 11272130 10.2337/diabetes.50.2.227

[26] Toshinai K (2003) Ghrelin-Induced Food Intake Is Mediated via the Orexin Pathway. Endocrinology 1506–1512. 12639935 10.1210/en.2002-220788

[27] Chen HY (2004) Orexigenic Action of Peripheral Ghrelin Is Mediated by Neuropeptide Y and Agouti-Related Protein. Endocrinology 2607–2612. 14962995 10.1210/en.2003-1596

[28] Kohno D, Gao HZ, Muroya S, Kikuyama S, Yada T (2003) Ghrelin directly interacts with neuropeptide-Y-containing neurons in the rat arcuate nucleus: Ca2+ signaling via protein kinase A and N-type channel-dependent mechanisms and cross-talk with leptin and orexin. Diabetes 948–956. 12663466 10.2337/diabetes.52.4.948

[29] Briggs DI, Enriori PJ, Lemus MB, Cowley MA, Andrews ZB (2010) Diet-Induced Obesity Causes Ghrelin Resistance in Arcuate NPY/AgRP Neurons. Endocrinology 4745–4755. 20826561 10.1210/en.2010-0556

[30] Seoane LM, Al-Massadi O, Pazos Y, Pagotto U, Casanueva FF (2006) Central obestatin administration does not modify either spontaneous or ghrelin-induced food intake in rats. J Endocrinol Invest RC13–15. 17033253 10.1007/BF03344174

[31] Nogueiras R, Pfluger P, Tovar S, Arnold M, Mitchell S, et al (2006) Effects of Obestatin on Energy Balance and Growth Hormone Secretion in Rodents. Endocrinology 21–26. 17008393 10.1210/en.2006-0915

[32] Hewson AK, Dickson SL (2000) Systemic administration of ghrelin induces Fos and Egr-1 proteins in the hypothalamic arcuate nucleus of fasted and fed rats. J Neuroendocrinol 12: 1047–1049. 11069119 10.1046/j.1365-2826.2000.00584.x

[33] Hinney A, Hoch A, Geller F, Schäfer H, Siegfried W, et al (2002) Ghrelin gene: identification of missense variants and a frameshift mutation in extremely obese children and adolescents and healthy normal weight students. Journal of Clinical Endocrinology & Metabolism 2716. 12050239 10.1210/jcem.87.6.8672

[34] Larsen L, Gjesing A, Sorensen T, Hamid Y, Echwald S, et al (2005) Mutation analysis of the preproghrelin gene: No association with obesity and type 2 diabetes. Clinical Biochemistry 420–424. 15820771 10.1016/j.clinbiochem.2005.01.008

[35] Steinle NI (2005) Variants in the Ghrelin Gene Are Associated with Metabolic Syndrome in the Old Order Amish. Journal of Clinical Endocrinology & Metabolism 6672–6677. 16204371 10.1210/jc.2005-0549

[36] Cellini E, Nacmias B, Brecelj-Anderluh M, Badía-Casanovas A, Bellodi L, et al (2006) Case-control and combined family trios analysis of three polymorphisms in the ghrelin gene in European patients with anorexia and bulimia nervosa. Psychiatr Genet 51–52. 16538179 10.1097/01.ypg.0000194444.89436.e9

[37] Kamegai J, Minami S, Sugihara H, Hasegawa O, Higuchi H, et al (1996) Growth hormone receptor gene is expressed in neuropeptide Y neurons in hypothalamic arcuate nucleus of rats. Endocrinology 137: 2109–2112. 8612554 10.1210/endo.137.5.8612554

[38] Li Y (2006) Mitochondrial aldehyde dehydrogenase-2 (ALDH2) Glu504Lys polymorphism contributes to the variation in efficacy of sublingual nitroglycerin. J Clin Invest 506–511. 16440063 10.1172/JCI26564 PMC1351000

[39] Mejias R, Adamczyk A, Anggono V, Niranjan T, Thomas GM, et al (2011) Gain-of-function glutamate receptor interacting protein 1 variants alter GluA2 recycling and surface distribution in patients with autism. Proceedings of the National Academy of Sciences 4920–4925. 10.1073/pnas.1102233108 PMC3064362 21383172

[40] Balthasar N, Mery PF, Magoulas CB, Mathers KE, Martin A, et al (2003) Growth hormone-releasing hormone (GHRH) neurons in GHRH-enhanced green fluorescent protein transgenic mice: a ventral hypothalamic network. Endocrinology 144: 2728–2740. 12746337 10.1210/en.2003-0006

[41] van den Pol AN, Yao Y, Fu LY, Foo K, Huang H, et al (2009) Neuromedin B and gastrin-releasing peptide excite arcuate nucleus neuropeptide Y neurons in a novel transgenic mouse expressing strong Renilla green fluorescent protein in NPY neurons. J Neurosci 29: 4622–4639. 19357287 10.1523/JNEUROSCI.3249-08.2009 PMC2745949

[42] Stengel A, Goebel M, Wang L, Rivier J, Kobelt P, et al (2010) Activation of brain somatostatin 2 receptors stimulates feeding in mice: analysis of food intake microstructure. Physiol Behav 101: 614–622. 20851136 10.1016/j.physbeh.2010.09.009 PMC2975782

[43] Wang L, Stengel A, Goebel M, Martinez V, Gourcerol G, et al (2011) Peripheral activation of corticotropin-releasing factor receptor 2 inhibits food intake and alters meal structures in mice. Peptides 32: 51–59. 20969907 10.1016/j.peptides.2010.10.017 PMC3010521

[44] Yu Y, South T, Huang XF (2009) Inter-meal interval is increased in mice fed a high whey, as opposed to soy and gluten, protein diets. Appetite 52: 372–379. 19100798 10.1016/j.appet.2008.11.011

[45] Steyn FJ, Huang L, Ngo ST, Leong JW, Tan HY, et al (2011) Development of a method for the determination of pulsatile growth hormone secretion in mice. Endocrinology 152: 3165–3171. 21586549 10.1210/en.2011-0253

